# Rethinking Oxidative Stress in *Helicobacter pylori*: A Multidisciplinary Framework Linking Redox Signaling, Cellular Reprogramming, and Diagnostic Precision in Gastric Carcinogenesis

**DOI:** 10.3390/life16060976

**Published:** 2026-06-09

**Authors:** Ayman Elbehiry, Adil Abalkhail, Sulaiman Anagreyyah, Suha Anjiria, Majed Aljahdali, Mamdouh Alharbi, Naif Almutairi, Mohammed Althagafi, Husam M. Edrees, Abdulaziz M. Almuzaini, Ihab M. Moussa, Saud Alhamyani, Abdulrahman Almaliki, Eman Marzouk

**Affiliations:** 1 Department of Public Health, College of Applied Medical Sciences, Qassim University, P.O. Box 6666, Buraydah 51452, Saudi Arabia; ar.elbehiry@qu.edu.sa (A.E.);; 2 Family Medicine Department, King Fahad Armed Forces Hospital, Jeddah 23311, Saudi Arabia; 3 Pharmacy Department, King Faisal Hospital, Makkah 24236, Saudi Arabia; 4 Medical Physics Department, Alhada Armed Forces Hospital, Taif 26792, Saudi Arabia; 5 Internal Medicine Department, King Fahad Armed Forces Hospital, Jeddah 23311, Saudi Arabia; 6 Laboratory Department, Armed Forces Center for Health Rehabilitation, Taif 21944, Saudi Arabia; 7 Department of Physiology, Faculty of Medicine, University of Tabuk, Tabuk 71491, Saudi Arabia; hedrees@ut.edu.sa; 8 Department of Veterinary Preventive Medicine, College of Veterinary Medicine, Qassim University, Buraydah 51452, Saudi Arabia; 9 Department of Botany and Microbiology, College of Science, King Saud University, P.O. Box 2455, Riyadh 11451, Saudi Arabia; 10 Specialist-Health Administration, Armed Forces Center for Health Rehabilitation, Taif 21944, Saudi Arabia; 11 Physiotherapy Department, Armed Forces Central for Health Rehabilitation, Taif 21944, Saudi Arabia

**Keywords:** *Helicobacter pylori*, gastric cancer, gastric carcinogenesis, virulence factors, chronic inflammation, immune dysregulation, oxidative stress, epigenetics, microbiome, signaling pathways

## Abstract

*Helicobacter pylori* (*H. pylori*) infection is one of the major causes of gastric cancer and remains an important global health concern. However, the biological processes linking chronic infection to malignant transformation are still not fully understood. Unlike previous reviews that mainly emphasized oxidative injury or individual virulence factors, this review synthesizes current evidence into an integrated redox-centered framework for gastric carcinogenesis. This framework links signaling pathways, epithelial adaptation, diagnostic interpretation, and therapeutic stratification. Current evidence indicates that persistent redox imbalance interacts with inflammatory signaling, mitochondrial dysfunction, metabolic reprogramming, epigenetic alteration, microbiome disruption, and oncogenic pathway activation throughout disease progression. Particular attention is given to the coordinated roles of NF-κB, STAT3, PI3K/AKT, HIF-1α, β-catenin, and Hippo-YAP signaling pathways. These pathways contribute to epithelial survival, chronic inflammation, genomic instability, and malignant transformation. This review additionally introduces a conceptual threshold model describing the progression from early epithelial stress to increasingly stable oncogenic reprogramming during long-standing infection. In addition, the limitations of conventional infection-centered diagnostics and non-selective antioxidant therapies are critically discussed. Emerging diagnostic approaches include oxidative injury biomarkers, transcriptomic and epigenetic profiling, artificial intelligence-assisted pathology, and multi-parameter predictive models. These approaches may improve risk stratification and facilitate earlier identification of high-risk gastric states. The translational implications further emphasize the importance of stage-specific and compartment-directed therapeutic strategies, particularly selective redox modulation and precision-guided targeting. Overall, this review provides a multidimensional perspective on *H. pylori*-associated gastric carcinogenesis and highlights future directions for predictive diagnostics, mechanistic stratification, and precision-based therapeutic development.

## 1. Introduction

Infection with *Helicobacter pylori* (*H. pylori*) is a major cause of gastric carcinogenesis and plays a central role in the progression from chronic gastritis to gastric adenocarcinoma [1]. Persistent colonization induces chronic inflammation that fails to eradicate the bacterium, resulting in prolonged immune activation and progressive mucosal injury [2]. This inflammatory environment promotes continuous production of reactive oxygen and nitrogen species by infiltrating immune cells and gastric epithelial cells, leading to chronic oxidative stress within the gastric mucosa [3].

Oxidative stress has long been recognized as an important link between *H. pylori* infection and gastric cancer because reactive species induce DNA damage, including oxidative base lesions such as 8-hydroxydeoxyguanosine, and alter cellular processes related to proliferation and apoptosis [4]. However, molecular injury alone does not fully explain the reproducible and multistep progression of gastric carcinogenesis. This limitation suggests that additional mechanisms are involved in maintaining and translating oxidative signals into stable cellular alterations [5,6].

Chronic infection maintains reactive oxygen and nitrogen species production through the combined activity of immune and epithelial cells involving NADPH oxidase (NOX1) and inducible nitric oxide synthase (iNOS) [7,8]. In addition to direct molecular damage, reactive species increasingly appear to function as regulators of intracellular signaling pathways associated with inflammation, survival, and epithelial adaptation [8,9].

Reactive oxygen species (ROS) influence epithelial turnover, inflammatory gene expression, and programmed cell death, while bacterial virulence factors such as *CagA* and *VacA* further disrupt host signaling and immune regulation [7,10].

Emerging evidence reveals that oxidative processes during *H. pylori* infection operate within interconnected signaling systems rather than as isolated cytotoxic events. Redox-associated signaling contributes to activation of pathways including nuclear factor kappa B (NF-κB), PI3K/AKT (phosphoinositide 3-kinase/protein kinase B), and Janus kinase/signal transducer and activator of transcription (JAK/STAT), which regulate inflammatory activity, cell survival, and proliferation [11].

Chronic infection is also associated with intracellular accumulation of reactive species and depletion of antioxidant defenses such as glutathione, reflecting a sustained imbalance in cellular redox homeostasis [10,12]. These changes influence epithelial behavior by linking oxidative signaling to both apoptosis and proliferative responses.

These findings support a broader view of oxidative stress in *H. pylori*-associated disease. Rather than functioning solely as a source of molecular injury, redox activity appears to participate in the regulation of cellular adaptation during chronic infection. This narrative review examines how redox-associated processes interact with inflammatory signaling, metabolic alteration, epigenetic remodeling, microbiome dynamics, and epithelial transformation during gastric carcinogenesis. It also explores the implications of these interactions for mechanistic interpretation, diagnostic development, and precision-guided therapeutic strategies in *H. pylori*-associated gastric cancer.

While *H. pylori* represents a major driver of gastric carcinogenesis, disease progression is recognized as multifactorial and may be influenced by additional environmental and host-related determinants [13]. Dietary patterns characterized by high salt intake, processed or smoked foods, reduced fruit and vegetable consumption, smoking, and behavioral exposures may interact with chronic inflammation and oxidative imbalance to modify epithelial adaptation and cancer susceptibility. These factors are therefore considered contextual modulators that may influence the biological consequences of persistent *H. pylori*-associated injury rather than independent alternatives to microbial pathogenesis [13,14,15].

**Figure 1:**
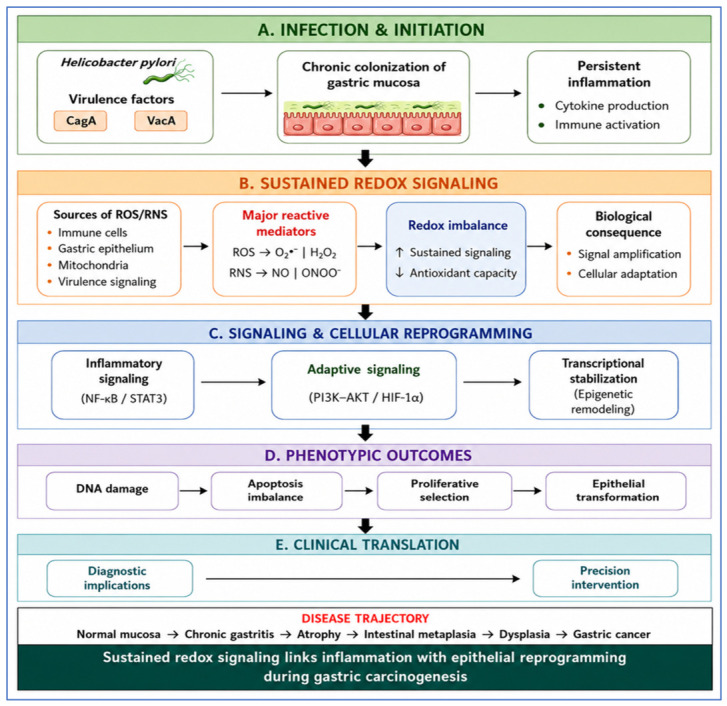
Redox-centered framework of *H. pylori*-associated gastric carcinogenesis. Chronic *H. pylori* infection induces sustained redox signaling that connects inflammatory activity with epithelial reprogramming across progressive disease states. These interactions are associated with altered cellular regulation, phenotypic transition, and movement toward clinically relevant gastric pathology.

Unlike previous reviews that primarily examined oxidative injury, inflammatory signaling, or individual molecular pathways separately, the present review integrates these processes within a unified redox-centered framework. In this model, redox regulation serves as a central link connecting epithelial adaptation, metabolic remodeling, epigenetic alteration, diagnostic interpretation, and therapeutic development [16,17]. The review also introduces a conceptual threshold model describing progressive biological transitions during chronic *H. pylori* infection and considers their potential diagnostic and translational implications. Figure 1 summarizes the proposed redox-centered model linking chronic *H. pylori* infection to progressive gastric carcinogenesis.

## 2. Literature Search Strategy

This narrative review was based on published studies examining the relationship between *H. pylori* infection, oxidative stress, redox signaling, inflammation, metabolic alterations, epigenetic regulation, and gastric carcinogenesis. Literature searches were conducted using PubMed, Scopus, and Web of Science for articles published up to April 2026. Search terms included “*H. pylori*”, “oxidative stress”, “redox signaling”, “reactive oxygen species”, “reactive nitrogen species”, “gastric cancer”, “gastric carcinogenesis”, “mitochondrial dysfunction”, “epigenetics”, “metabolic reprogramming”, “NF-κB”, “STAT3”, “PI3K/AKT”, “microbiome”, “biomarkers”, and “precision therapy”, used individually and in relevant combinations.

Inclusion criteria comprised original experimental studies, clinical investigations, translational studies, systematic reviews, meta-analyses, and high-quality narrative reviews that provided mechanistic, diagnostic, prognostic, or therapeutic insights related to *H. pylori*-associated gastric carcinogenesis. Exclusion criteria included studies not directly related to gastric carcinogenesis, non-English publications, conference abstracts without full-text availability, and duplicate reports.

Studies were identified through title, abstract, and full-text evaluation and were selected according to their relevance to the topics addressed in this review. Because this work represents a narrative review, no formal quality-scoring system or quantitative evidence synthesis was performed. The selected literature was critically integrated to provide a comprehensive overview of the biological mechanisms, diagnostic approaches, and therapeutic implications associated with *H. pylori*-driven gastric carcinogenesis.

## 3. Redox Biology as a Regulatory System in *H. pylori* Infection

### 3.1. Sustained Redox Regulation and Adaptive Signaling During Chronic Infection

Chronic *H. pylori* infection maintains continuous production of reactive oxygen and nitrogen species through coordinated activity of infiltrating immune cells and gastric epithelial cells. Activated neutrophils and macrophages generate superoxide and hydrogen peroxide through NOX1-associated mechanisms, whereas epithelial cells further contribute to intracellular oxidative activity following bacterial stimulation [7,18]. Inducible nitric oxide synthase (iNOS) is also upregulated in both immune and epithelial compartments, increasing nitric oxide production and promoting formation of reactive nitrogen intermediates such as peroxynitrite through interaction with superoxide [19,20]. In addition, *H. pylori* enhances oxidative activity by disrupting phagocytic regulation and amplifying epithelial NOX1-associated signaling [21].

Additional bacterial factors may further contribute to oxidative and inflammatory responses during chronic infection. *H. pylori* neutrophil-activating protein (NAP) promotes recruitment and activation of neutrophils and monocytes, enhancing production of reactive oxygen intermediates and pro-inflammatory cytokines within the gastric mucosa [22,23]. Heat shock protein-associated responses have likewise been implicated in immune activation and oxidative signaling through interactions with host stress-response pathways [21]. These mechanisms may further amplify local oxidative injury and inflammatory signaling during persistent infection.

Unlike acute inflammatory responses characterized by transient oxidative bursts and rapid resolution, chronic infection produces prolonged low-to-moderate reactive species exposure that remains compatible with epithelial survival. Under these conditions, oxidative activity increasingly functions as a regulator of intracellular signaling and tissue behavior rather than solely as a source of direct molecular injury [24,25]. Overall, current evidence supports a transition from short-lived oxidative injury toward a relatively stable redox-associated regulatory environment during persistent infection.

Continuous exposure to reactive intermediates progressively alters intracellular redox balance within gastric epithelial cells. Chronic infection is associated with ROS accumulation together with depletion of reduced glutathione, indicating altered intracellular redox balance without widespread cell death [12,26].

At the tissue level, these changes are accompanied by altered antioxidant enzyme activity, including superoxide dismutase and catalase, together with increased oxidative stress markers within the gastric mucosa [9,27]. These findings suggest establishment of a stable redox state in which oxidative generation and antioxidant buffering remain altered while preserving cellular viability [12].

Mitochondria contribute substantially to maintenance of this redox state. Persistent infection promotes continuous mitochondrial ROS production together with incomplete functional recovery, thereby sustaining intracellular conditions favorable for prolonged signaling activity [9,28]. This redox imbalance influences epithelial proliferation, apoptosis regulation, and cellular stress responses during chronic infection. These observations support the concept that long-standing *H. pylori* colonization establishes a sustained redox-associated state that extends beyond acute host defense and contributes to long-term biological regulation within the gastric mucosa.

### 3.2. Spatial Organization and Adaptive Consequences of Redox Signaling

The biological effects of reactive species during chronic *H. pylori* infection depend not only on their abundance but also on their intracellular localization and duration of exposure. Distinct production sites, including the plasma membrane, mitochondria, and immune-cell interfaces, generate compartment-specific oxidative signals capable of influencing different cellular processes [9,29]. At the membrane level, activation of pattern-recognition receptors such as TLR4 promotes localized oxidative signaling linked to downstream inflammatory responses [29]. Mitochondrial oxidative activity is additionally enhanced by bacterial virulence factors including *CagA*, which disrupt mitochondrial integrity and promote intracellular stress signaling [9].

Temporal characteristics further influence the biological consequences of oxidative exposure. Chronic low-level reactive species generation promotes repeated activation of redox-sensitive signaling systems over extended periods, whereas short-term high-intensity exposure is more commonly associated with acute cellular injury [11]. The biological impact of oxidative activity therefore depends not only on peak intensity, but also on sustained signaling behavior and persistent intracellular exposure. These observations support the view that reactive intermediates function as spatially and temporally organized signaling mediators during chronic infection rather than isolated cytotoxic byproducts [17].

Gastric epithelial cells progressively adapt to these chronic oxidative conditions through coordinated changes involving mitochondrial activity, metabolism, and stress-response behavior. Although early compensatory mechanisms may initially limit cellular injury, prolonged oxidative exposure gradually promotes accumulation of dysfunctional mitochondria, impaired oxidative phosphorylation, and sustained intracellular reactive oxygen species production [10,30]. Concurrently, infected epithelial cells increasingly rely on glycolytic metabolism, reflecting reduced dependence on mitochondrial oxidative phosphorylation under chronic oxidative pressure [28].

Despite these alterations, antioxidant systems remain partially functional, allowing epithelial cells to tolerate persistent oxidative conditions while preserving responsiveness to redox-associated signaling [12]. This adaptive state supports epithelial survival during chronic infection and provides the foundation for the inflammatory, metabolic, and transcriptional changes discussed in subsequent sections.

To distinguish sustained redox-associated regulation from classical acute oxidative injury, Table 1 summarizes their principal mechanistic and biological differences in the context of chronic *H. pylori* infection.

This comparison highlights the distinction between transient oxidative injury and sustained redox-associated regulation during chronic *H. pylori* infection and provides the mechanistic foundation for the adaptive epithelial changes discussed in the following sections.

## 4. Redox-Integrated Cellular Reprogramming

Persistent redox imbalance during chronic *H. pylori* infection alters epithelial behavior through interconnected effects on inflammatory regulation, cellular metabolism, chromatin organization, and host-microbial interactions. Rather than producing isolated molecular alterations, prolonged oxidative exposure promotes adaptive changes that modify tissue organization and epithelial function [9,17]. These responses support survival under chronic inflammatory conditions while promoting loss of normal epithelial regulation.

### 4.1. Inflammatory and Metabolic Adaptation

Long-standing *H. pylori* infection promotes continuous recruitment of immune cells together with sustained cytokine production within the gastric mucosa [22,31]. This persistent inflammatory environment alters epithelial behavior through repeated activation of stress-responsive signaling pathways and inflammatory mediators associated with tissue remodeling [32,33]. Bacterial virulence factors further intensify these responses through direct modulation of host–cell regulation. *CagA* enhances inflammatory signaling and NOX1-associated oxidative activity, whereas pattern-recognition receptor activation promotes additional cytokine release and immune stimulation [28,34].

Interactions among epithelial cells, infiltrating immune populations, and inflammatory mediators favor epithelial states associated with enhanced survival, altered turnover, and impaired restoration of mucosal integrity [1,33]. Consequently, the inflammatory microenvironment shifts from a protective response toward a chronically dysregulated state that supports epithelial adaptation during persistent infection [1,32].

Chronic oxidative exposure is accompanied by metabolic restructuring that supports epithelial survival under altered conditions. *H. pylori* infection promotes increased glycolytic activity together with alterations in lipid and amino acid metabolism, generating a metabolic profile adapted to sustained cellular stress [35]. Increased glycolytic flux is associated with upregulation of glucose transport systems and metabolic enzymes as well as stabilization of hypoxia-inducible factor-1 alpha (HIF-1α), which promotes transcription of glycolysis-associated genes [36,37]. Mitochondrial oxidative phosphorylation becomes less efficient, shifting ATP production toward glycolytic pathways [28].

Additional metabolic alterations further contribute to epithelial adaptation. Changes in lipid metabolism support membrane synthesis and intracellular regulatory activity, whereas enhanced glutamine utilization influences nutrient sensing and growth-associated signaling pathways including mTORC1 [35,38]. Metabolic products such as lactate additionally influence immune-cell behavior and may contribute to persistence of the altered gastric microenvironment during chronic infection [38].

Recent evidence further suggests that metabolic remodeling may influence ferroptosis-associated responses during gastric epithelial transformation [39]. These changes support epithelial viability under conditions of prolonged oxidative and metabolic stress. These metabolic adaptations extend beyond cellular energetics and may contribute to more durable alterations in gene regulation and epithelial behavior during chronic infection.

### 4.2. Epigenetic and Microbiome-Associated Remodeling

Sustained inflammatory and metabolic alterations during chronic infection are accompanied by epigenetic remodeling involving DNA methylation, histone-associated modification, and altered non-coding RNA expression [40,41]. These changes influence transcriptional regulation of genes associated with epithelial differentiation, proliferation, genomic maintenance, and cellular survival [42,43,44].

Oxidative conditions affect the activity of DNA methyltransferases and chromatin-associated regulatory enzymes, thereby promoting accumulation of stable epigenetic alterations over time [43,44]. Altered microRNA expression additionally contributes to maintenance of abnormal transcriptional programs linked to inflammatory and proliferative activity [45]. Unlike transient signaling responses, these chromatin-associated modifications may persist despite changes in inflammatory intensity, allowing long-term maintenance of altered epithelial phenotypes [41,43].

Epigenetic remodeling provides a mechanism through which chronic inflammatory and metabolic alterations become stabilized within gastric epithelial populations, thereby facilitating long-term dysregulation during gastric carcinogenesis [41,44].

Chronic *H. pylori* infection additionally changes gastric microbial composition and reduces microbial diversity, favoring expansion of dysbiosis-associated microbial communities linked to altered inflammatory and metabolic activity [46,47]. Microbial metabolites including lactate, short-chain fatty acids, and nitrogen-associated intermediates further contribute to alterations in epithelial metabolism and mucosal homeostasis [48,49].

Changes in gastric pH, mucosal integrity, and nutrient availability additionally facilitate colonization by other microbial species, thereby reshaping immune interactions within the gastric environment [50]. Metagenomic and metabolomic investigations further demonstrate associations between microbial alterations and pathways related to energy metabolism and oxidative injury [49]. These ecological changes therefore appear to reinforce epithelial and metabolic alterations associated with long-standing *H. pylori* colonization.

**Figure 2:**
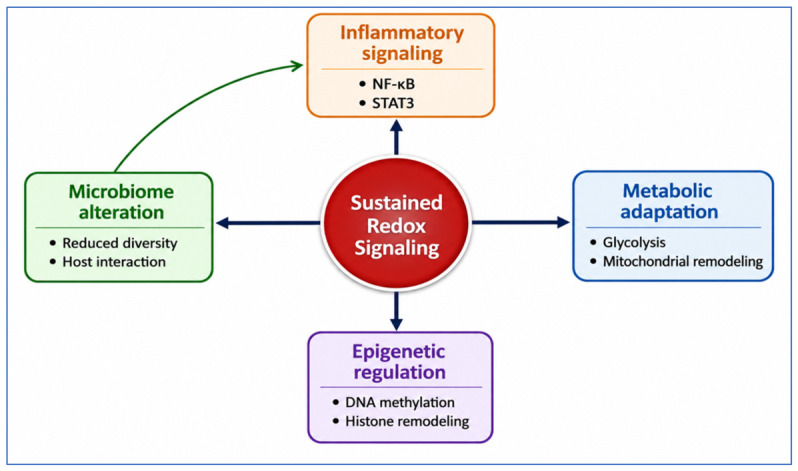
Integrated interaction network during chronic *H. pylori* infection. Sustained redox signaling is presented as a shared biological interface connecting inflammatory signaling, metabolic adaptation, epigenetic regulation, and microbiome-associated alteration. The framework illustrates how these interconnected processes may reinforce persistent cellular imbalance during chronic gastric injury.

Figure 2 summarizes the interconnected relationship among inflammatory remodeling, metabolic adaptation, epigenetic alteration, and microbiome-associated dysregulation during chronic *H. pylori* infection.

## 5. Redox Governance of Oncogenic Signaling

The adaptive cellular changes described in the preceding section provide the biological context for activation of signaling networks that progressively reshape epithelial behavior during disease progression.

### 5.1. Redox-Responsive Signaling Networks and Genomic Destabilization

Chronic *H. pylori* infection establishes a persistent inflammatory environment that promotes epithelial survival despite ongoing cellular injury. Sustained oxidative and cytokine-associated signaling alters transcriptional activity within gastric epithelial cells, favoring proliferation, apoptosis resistance, and tissue remodeling [23,51].

NF-κB activation promotes expression of inflammatory mediators including interleukin-8 and cyclooxygenase-2, thereby sustaining immune-cell recruitment within the gastric mucosa [52,53]. Concurrently, cytokine-associated STAT3 activation supports epithelial persistence through regulation of survival- and proliferation-associated genes [54]. Bacterial virulence factors further intensify these responses. *CagA* enhances inflammatory and proliferative signaling through Src- and ERK-associated mechanisms, whereas *VacA* disrupts mitochondrial integrity and modifies epithelial and immune-cell behavior [34,55]. Toll-like receptor activation additionally amplifies inflammatory signaling through MyD88-dependent pathways [20].

Crosstalk among NF-κB, STAT3, and HIF-1α links inflammatory signaling with metabolic adaptation and epithelial survival during chronic infection [28,37,56]. HIF-1α stabilization promotes transcriptional responses associated with glycolytic metabolism and stress adaptation, whereas STAT3 regulates mediators including BCL-XL and survivin that preserve epithelial viability during chronic infection [37,56]. Crosstalk involving β-catenin- and Hippo-YAP-associated signaling supports proliferation, epithelial plasticity, and tissue remodeling within the gastric mucosa [1,57]. These pathways disrupt epithelial regulation and reinforce carcinogenic remodeling within the gastric mucosa [17,32]. Sustained activation of these signaling pathways not only promotes epithelial survival but also contributes to progressive genomic instability during chronic infection.

Chronic oxidative and nitrosative injury compromises genomic stability within gastric epithelial cells. Chronic infection promotes accumulation of oxidative DNA lesions including 8-hydroxy-2′-deoxyguanosine (8-OHdG), strand breaks, and replication-associated stress, thereby increasing mutational burden within gastric epithelium [58,59]. Inflammatory-cell infiltration further intensifies genomic injury through continuous production of nitric oxide and superoxide, which generate highly reactive intermediates capable of damaging both nuclear and mitochondrial DNA [60].

Mitochondrial genomes are particularly vulnerable because of their proximity to the electron transport chain and limited structural protection. Experimental studies demonstrate associations among *H. pylori* infection, mitochondrial DNA mutation, and altered mitochondrial genome maintenance [57,61]. DNA repair capacity also declines during prolonged infection. Reduced expression of mismatch-repair and double-strand repair proteins including MLH1, MGMT, MSH2, and MRE11 limits correction of accumulated genomic injury [57,62,63]. Promoter hypermethylation further suppresses several repair-associated genes [57].

Checkpoint regulation is likewise altered during chronic infection. PI3K/AKT- and ERK-associated signaling promotes continued cell-cycle progression despite unresolved DNA injury, thereby weakening surveillance mechanisms and increasing chromosomal instability [34,64]. Oxidative conditions additionally interfere with p53-associated responses, reducing elimination of damaged epithelial cells [57]. Additional contributors including spermine oxidase-dependent hydrogen peroxide production and mitochondrial dysfunction further contribute to genomic destabilization during chronic infection [28,65]. These alterations promote accumulation of genomic abnormalities during gastric carcinogenesis.

### 5.2. Epigenetic Remodeling and Oncogenic Network Convergence

Persistent inflammatory and oxidative signaling promotes durable transcriptional dysregulation through epigenetic remodeling. Oxidative conditions influence DNA methyltransferases, chromatin-associated regulatory enzymes, and histone-modifying systems, thereby producing durable alterations in gene regulation that extend beyond transient inflammatory stimulation [66,67].

Promoter hypermethylation affects multiple tumor suppressor and DNA repair genes including *CDH1*, *RUNX3*, *MLH1*, *MGMT*, *APC*, and *PTEN* [40,67]. Increased methyltransferase activity together with accumulation of methylated CpG islands reinforces transcriptional silencing within gastric epithelial cells [66,68]. Histone-associated modification alters chromatin accessibility and favors transcriptional states linked to proliferation, epithelial transition, and apoptosis resistance [69]. These changes further impair genomic maintenance and contribute to cellular instability.

Altered non-coding RNA expression provides an additional layer of regulatory disruption during chronic infection. Changes in microRNA-associated signaling influence inflammatory activity, epithelial proliferation, therapeutic responsiveness, and survival behavior [45,67]. Unlike transient inflammatory signaling, these epigenetic alterations may persist despite changing inflammatory conditions and continue to support malignant cellular behavior [40,67]. These persistent transcriptional alterations create a permissive environment for coordinated activation of oncogenic signaling networks during malignant progression.

Malignant transformation during chronic *H. pylori* infection arises through coordinated interaction among multiple signaling pathways rather than isolated molecular events. NF-κB, STAT3, PI3K/AKT, MAPK, HIF-1α, β-catenin, and Hippo-YAP collectively influence inflammatory persistence, epithelial survival, metabolic adaptation, genomic instability, and tissue remodeling during chronic infection [34,70].

Within this integrated network, HIF-1α-associated responses link inflammatory signaling with altered cellular metabolism, whereas PI3K/AKT and MAPK pathways sustain epithelial survival and proliferative activity [37,71]. β-catenin and Hippo-YAP signaling further contribute to epithelial plasticity, invasive behavior, and stemness-associated functions during malignant progression [1,57]. Cytokines, immune-cell infiltration, metabolic products, and microbiome-associated mediators sustain these signaling responses within the gastric mucosa [48,49].

These pathways promote impaired growth control, genomic instability, and sustained oncogenic signaling during gastric carcinogenesis. Table 2 summarizes the principal redox-responsive pathways involved in inflammatory activity, epithelial survival, genomic instability, and malignant transformation during chronic *H. pylori* infection.

## 6. Mechanistic Threshold Framework of Gastric Carcinogenesis

This threshold framework is presented as a conceptual model integrating the biological alterations associated with chronic *H. pylori* infection rather than a clinically validated staging system. The transition states are intended to organize progressive biological changes and should not be interpreted as categories defined by specific molecular, histological, or clinical criteria. Gastric carcinogenesis develops through overlapping inflammatory, metabolic, genomic, and epigenetic alterations that accumulate during prolonged infection. Within this framework, the following thresholds describe biologically distinct phases associated with declining epithelial regulation and reduced reversibility during chronic infection.

### 6.1. Initiation Threshold

The initiation phase begins when oxidative injury exceeds the compensatory capacity of gastric epithelial cells and disrupts normal regulatory function. At this stage, oxidative exposure shifts from a transient inflammatory response toward a sustained source of epithelial injury [9,28].

Early alterations may include depletion of antioxidant buffering systems, incomplete mitochondrial recovery, and accumulation of oxidative DNA lesions before overt structural transformation becomes evident [9,12]. Bacterial virulence factors including *CagA* and *VacA* intensify these disturbances through mitochondrial injury, epithelial stress signaling, and prolonged mucosal stimulation [1,34,72]. Repeated inflammatory injury weakens recovery of normal gastric epithelial function.

Although epithelial architecture remains largely preserved during this phase, cellular recovery becomes incomplete and favors continued molecular instability within infected tissue [19]. The initiation threshold reflects an early shift toward altered epithelial regulation during chronic infection.

### 6.2. Adaptation Threshold

Persistent inflammatory and metabolic stress favors survival of epithelial-cell populations capable of functioning under chronically altered conditions. During this stage, epithelial responses shift from temporary stress adaptation toward more stable survival-associated phenotypes [9,17].

This adaptive transition is accompanied by altered nutrient utilization, metabolic flexibility, and preservation of proliferative activity despite impaired mitochondrial efficiency [35,37]. Glycolytic metabolism becomes more prominent together with HIF-1α-associated metabolic regulation and altered glucose, lipid, and amino acid utilization [35,36]. Concurrent epigenetic remodeling involving DNA methylation, histone-associated regulation, and altered microRNA expression further stabilizes transcriptional programs associated with epithelial survival under chronic stress conditions [43,44].

Local tissue conditions further support this adaptive environment. Cytokines, infiltrating immune populations, and microbiome-associated metabolites influence epithelial selection pressures and favor cell populations capable of tolerating chronic cellular stress [48,49]. The adaptation threshold therefore reflects selection of epithelial populations capable of maintaining viability despite ongoing inflammatory and metabolic injury.

### 6.3. Dominance Threshold

The dominance phase emerges when proliferative and survival-associated signaling overrides genomic surveillance and growth regulation. During this phase, epithelial expansion persists despite unresolved DNA injury, checkpoint dysfunction, and accumulation of molecular abnormalities [57,73].

Inflammatory and survival-associated signaling pathways including NF-κB and STAT3 contribute to maintenance of sustained growth activity, apoptosis resistance, and persistent inflammatory amplification within the gastric mucosa [56,74]. Impaired mismatch-repair activity together with replication-associated stress further increases genomic instability within epithelial populations [59,62,75].

Additional signaling systems including β-catenin and Hippo-YAP further support epithelial plasticity, invasive behavior, and stemness-associated characteristics during chronic infection [57,76]. At this stage, regulatory control weakens as inflammatory mediators and proliferative signaling pathways reinforce one another despite fluctuating tissue conditions [51]. The dominance threshold reflects transition toward self-sustaining oncogenic behavior characterized by impaired growth regulation and persistent genomic instability.

### 6.4. Commitment Threshold

The commitment phase emerges when malignant cellular behavior persists despite reduced dependence on the initiating inflammatory environment. During this stage, genomic instability, metabolic alteration, mitochondrial dysfunction, and epigenetic remodeling support maintenance of malignant cellular phenotypes [77,78].

Promoter hypermethylation affecting tumor suppressor and DNA repair genes including CDH1, RUNX3, MLH1, and MGMT accumulates within epithelial populations [40,42]. Histone-associated remodeling and non-coding RNA dysregulation further maintain transcriptional programs linked to proliferation, apoptosis resistance, epithelial transition, and invasive behavior [41,44]. Genomic integrity is normally maintained through coordinated DNA repair mechanisms, including base-excision repair of oxidative DNA lesions, mismatch repair of replication-associated errors, and double-strand break repair pathways. Defects involving these repair systems additionally contribute to genomic instability during malignant progression [57,59,63].

These transition states are intended to represent overlapping biological tendencies rather than discrete histopathological stages [51]. Epithelial populations display autonomous proliferation, altered chromatin organization, persistent genomic instability, and reduced responsiveness to normal growth restriction.

**Figure 3:**
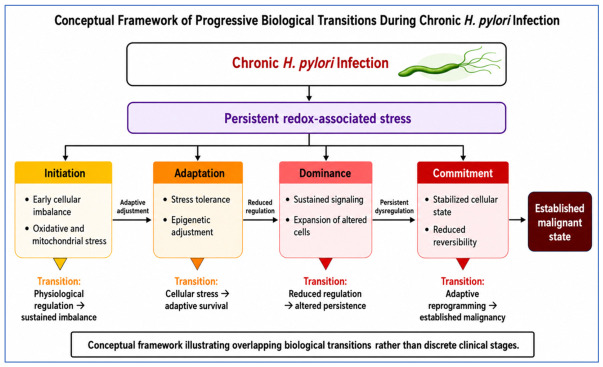
Conceptual framework of progressive biological transitions during chronic *H. pylori* infection. Persistent redox-associated stress is presented as a potential integrating process linking sequential but overlapping changes in cellular behavior. The framework illustrates a gradual shift from early imbalance toward progressively stabilized pathological states across chronic gastric injury.

The commitment threshold therefore represents transition toward established *H. pylori*-associated malignancy characterized by durable oncogenic regulation and limited functional reversibility [77,78]. Figure 3 illustrates the proposed progression from early intracellular imbalance toward progressively stabilized malignant transformation during chronic *H. pylori* infection.

## 7. The Antioxidant Paradox as a Systems-Level Limitation

These mechanistic insights have important therapeutic implications and help explain the restricted success of broadly applied antioxidant strategies in chronic disease settings.

### 7.1. Limitations of Broad Antioxidant Strategies

Although oxidative imbalance is strongly associated with *H. pylori*-related gastric carcinogenesis, generalized antioxidant approaches have produced inconsistent clinical outcomes. Experimental evidence consistently links reactive-species accumulation with inflammatory injury, genomic instability, and epithelial transformation, yet broad antioxidant supplementation has not reliably prevented malignant progression or produced durable therapeutic benefit [9,12].

Clinical responses appear highly dependent on disease stage, cellular localization, tissue context, and intensity of oxidative exposure rather than simple reduction in reactive intermediates alone [16,17]. In some settings, antioxidant administration partially reduces tissue injury, whereas in others it produces limited long-term biological improvement despite measurable reductions in oxidative markers.

These findings illustrate that reactive oxygen and nitrogen species cannot be interpreted exclusively as harmful byproducts during chronic *H. pylori* infection. Oxidative activity additionally participates in epithelial maintenance, immune coordination, and adaptive stress regulation, thereby making indiscriminate suppression biologically unpredictable rather than uniformly protective [79].

Reactive intermediates contribute to multiple processes required for normal cellular organization, including kinase regulation, mitochondrial communication, transcriptional control, and intercellular signaling [9,37]. Gastric epithelial cells therefore depend on tightly regulated redox activity to maintain normal cellular function under both physiological and inflammatory conditions.

During chronic infection, compensatory antioxidant systems partially restrict excessive intracellular injury while preserving signaling processes required for epithelial adaptation. Glutathione-associated pathways, peroxiredoxin 2 (PRDX2), and related antioxidant mechanisms contribute to maintenance of redox balance despite chronic inflammatory exposure [12,80].

Experimental investigations additionally demonstrate that complete suppression of reactive-species signaling may impair cellular coordination and disrupt adaptive responses necessary for epithelial survival [9,12]. These results support the concept that oxidative signaling functions not only as a mediator of injury, but also as an essential component of intracellular regulation during chronic infection. These observations indicate that effective intervention requires preservation of physiological redox signaling while limiting pathological oxidative activity associated with disease progression.

Non-specific antioxidant approaches may simultaneously inhibit pathological oxidative activity and physiologically necessary signaling processes. Because reactive intermediates influence immune defense, epithelial repair, mitochondrial function, and stress adaptation, broad suppression can disrupt cellular regulation rather than restore normal tissue behavior [9,12].

This limitation becomes particularly relevant during chronic *H. pylori* infection, where oxidative activity contributes both to carcinogenic progression and to adaptive responses that partially restrict uncontrolled cellular injury. Experimental studies suggest that excessive suppression may alter epithelial stress tolerance, disrupt immune coordination, and modify survival behavior without fully interrupting oncogenic signaling networks [9,80].

Accordingly, generalized antioxidant administration may produce biological imbalance rather than durable protection. Failure of these approaches appears to reflect insufficient mechanistic selectivity rather than lack of therapeutic relevance of redox-associated pathways themselves [16,17]. Table 3 summarizes the principal system-level limitations associated with non-selective antioxidant intervention during chronic *H. pylori*-associated carcinogenesis.

### 7.2. Precision Redox Modulation as a Therapeutic Perspective

Current evidence increasingly supports selective regulation of defined oxidative pathways rather than indiscriminate antioxidant administration. Effective intervention will likely depend on targeting specific signaling systems, intracellular compartments, or temporally restricted oxidative processes associated with malignant progression [1,17].

Potential approaches include modulation of mitochondrial oxidative activity, NF-κB-associated signaling, antioxidant-response pathways, and compartment-specific signaling interactions linked to epithelial transformation and inflammatory persistence. Experimental studies have proposed that nanotherapeutic delivery systems and pathway-selective interventions may improve targeting precision while preserving physiological cellular organization [81,82].

Recent mechanistic studies further demonstrate that pathway-directed oxidative manipulation has been explored experimentally against *H. pylori* itself, emphasizing the importance of targeted therapeutic design [83].

The antioxidant paradox does not indicate failure of redox biology as a therapeutic concept. Instead, it reflects the limitations of non-selective antioxidant strategies that cannot adequately distinguish pathological oxidative injury from essential intracellular signaling functions [9].

## 8. Diagnostic Innovation and Contextual Performance Evaluation

The biological complexity outlined in previous sections also highlights the need for diagnostic approaches that extend beyond simple detection of infection.

### 8.1. Limitations of Detection-Centered Diagnostics

Most currently used diagnostic methods for *H. pylori* infection are designed primarily to confirm bacterial presence rather than evaluate the biological consequences of chronic infection within gastric tissue [84,85]. Techniques including urea breath testing, stool antigen assays, serology, histopathology, and molecular detection provide important information regarding infection status, yet they offer limited insight into mucosal instability or future disease behavior [86].

This limitation becomes particularly important during long-standing infection because individuals with comparable colonization levels may develop markedly different clinical outcomes. Some patients remain relatively stable, whereas others progress toward atrophy, intestinal metaplasia, dysplasia, or gastric carcinoma despite similar microbiological findings [16,87].

Several commonly used diagnostic methods demonstrate high analytical performance for detecting active infection. Urea breath testing and stool antigen assays typically achieve sensitivity and specificity values exceeding 90% under appropriate clinical conditions [85,88]. However, these approaches primarily confirm bacterial persistence and provide limited information regarding epithelial remodeling, genomic instability, or malignant susceptibility.

Thus, infection detection alone provides insufficient prognostic resolution. Clinically meaningful evaluation increasingly requires assessment of inflammatory activity, oxidative injury, tissue remodeling, and early epithelial change rather than simple confirmation of bacterial colonization [87,89].

### 8.2. Biomarkers Reflecting Active Mucosal Alteration

Emerging diagnostic approaches increasingly focus on tissue-associated changes linked to active disease progression. Oxidative DNA injury products, inflammatory mediators, mitochondrial stress markers, and transcription-associated abnormalities provide insight into ongoing mucosal disruption during chronic infection [3,66,90].

Accumulation of 8-hydroxy-2′-deoxyguanosine (8-OHdG) has been associated with inflammatory severity, cagA-positive strains, intestinal metaplasia, and gastric carcinogenesis, supporting its potential value as an indicator of active genomic injury [3,90]. Additional markers including malondialdehyde, IL-8, total oxidant status, and altered pepsinogen ratios may further assist in distinguishing relatively stable colonization from progressive mucosal deterioration [23,91].

Recent advances in transcriptomic profiling, epigenetic analysis, and non-coding RNA characterization additionally improve evaluation of tissue-level regulatory dysregulation [66,92]. Combined interpretation of oxidative, inflammatory, histological, and transcription-associated findings may enhance recognition of clinically significant gastric abnormalities before irreversible structural disruption becomes established [89,90].

### 8.3. Interpreting Diagnostic Performance in Biological Context

Diagnostic performance in *H. pylori*-associated disease extends beyond analytical confirmation of infection alone. High sensitivity may accurately identify bacterial colonization while providing limited information regarding epithelial instability or future malignant potential [86,93]. Similarly, specificity requires broader interpretation than discrimination between infected and non-infected individuals because clinically relevant assessment increasingly depends on identification of mucosal states associated with atrophy, intestinal metaplasia, genomic injury, dysplasia, and early neoplastic transformation [87,91].

Single biomarkers often provide limited predictive precision when interpreted independently. Histopathological evaluation remains important for identifying structural alterations including atrophy and intestinal metaplasia, whereas molecular and inflammatory biomarkers may better reflect dynamic biological activity [16,87]. Pepsinogen profiling has additionally demonstrated utility in identifying gastric atrophy and elevated malignant susceptibility, particularly when integrated with histological and inflammatory findings [91,94].

Combined assessment incorporating histopathological features, oxidative injury indicators, inflammatory mediators, and transcription-associated findings appears more informative than isolated testing strategies alone [87,91]. Table 4 summarizes the principal diagnostic approaches currently used in *H. pylori*-associated disease together with their analytical performance considerations, biological relevance, and limitations in evaluating gastric carcinogenic progression.

### 8.4. Transition Toward Predictive Diagnostic Models

Future diagnostic strategies will likely depend on integrated interpretation of histological, inflammatory, genomic, and tissue-associated information rather than isolated testing methods. Evaluation of oxidative DNA injury, cytokine profiles, genomic instability, pepsinogen status, and mucosal architecture may support recognition of patients with increased susceptibility to gastric malignancy during chronic *H. pylori* infection [89,94].

Computational approaches increasingly facilitate earlier identification of high-risk gastric states. Integration of transcriptomic signatures, epigenetic profiles, non-coding RNA patterns, and histopathological features may improve recognition of clinically significant tissue changes before advanced structural damage becomes apparent [92].

Artificial intelligence (AI)-assisted histopathological analysis and machine learning-based biomarker interpretation additionally show promising potential for risk stratification of progressive gastric lesions. Emerging predictive models integrating tissue architecture, inflammatory activity, and molecular alterations have reported preliminary improvements in diagnostic assessment for identifying atrophy, intestinal metaplasia, and early neoplastic transformation [95,96]. Although several predictive platforms are currently investigational, early findings support their potential utility for risk stratification and surveillance planning. Quantitative performance estimates for emerging biomarkers, AI-assisted pathology, and predictive models remain heterogeneous across studies and are not yet sufficiently standardized for direct comparison, highlighting the need for further prospective validation before routine clinical implementation [95,96].

These developments support a transition from static infection confirmation toward biologically contextualized evaluation of disease behavior. Such predictive approaches may support individualized surveillance, timely clinical intervention, and improved assessment of gastric cancer susceptibility during chronic *H. pylori*-associated disease [97,98].

Importantly, the proposed diagnostic framework is not intended to replace current *H. pylori* screening and eradication strategies, which remain central components of gastric cancer prevention [16]. Rather, this perspective recognizes that infection status alone may not fully capture long-term biological consequences in all individuals, particularly after prolonged exposure or following microbial eradication [67]. Integrating biological markers with infection assessment may therefore support improved identification of individuals who remain at elevated risk despite successful eradication.

**Table 4:** Comparative diagnostic performance and biological interpretation of currently used methods for *H. pylori* detection.

Diagnostic Method	Biological Target	Representative Diagnostic Performance	Clinical Considerations	Biological Relevance Beyond Infection Detection	Refs.
^13^C Urea Breath Test (UBT)	Active urease activity	Sensitivity 94% at fixed specificity 90%	High comparative accuracy for active infection; suitable for diagnosis and post-eradication confirmation	Confirms active infection but does not evaluate epithelial transformation or malignant risk	[88]
^14^C Urea Breath Test (UBT)	Active urease activity	Sensitivity 92% at fixed specificity 90%	Reliable assessment of active infection	Limited value for biological risk interpretation	[88]
Stool Antigen Test (SAT)	Bacterial antigen detection	Diagnostic performance varies according to assay platform and threshold selection	Useful for diagnosis and follow-up using validated assays	Reflects infection status without evaluating progression-related tissue alterations	[85,88,99]
Serological Assays	Host antibody response	Sensitivity 84% at fixed specificity 90%	Useful for exposure assessment but unable to distinguish active from previous infection	Reflects immune exposure rather than current mucosal activity	[85,88,99]
Histopathology	Tissue architecture and bacterial visualization	Sensitivity approximately 91–93%; specificity up to 100% under optimized conditions	Allows direct tissue interpretation with observer and sampling variability	Identifies atrophy, intestinal metaplasia, dysplasia, and inflammatory remodeling	[84,85]
PCR-based Molecular Assays	Bacterial DNA and resistance-associated markers	Sensitivity approximately 95%; specificity approximately 95% (method dependent)	Enables molecular characterization and resistance profiling	Supports virulence assessment but provides limited direct information on tissue behavior	[84]

Footnote: Diagnostic performance values represent estimates extracted from systematic reviews and diagnostic review articles and should not be interpreted as fixed universal values because methodology, reference standards, medication exposure, sampling conditions, and study populations differ across studies.

## 9. Translational and Therapeutic Implications

Advances in mechanistic understanding and diagnostic stratification provide an important foundation for development of more targeted therapeutic approaches.

### 9.1. Persistence of Biological Alteration After Eradication

Eradication of *H. pylori* substantially reduces gastric cancer risk but does not fully reverse many tissue-associated abnormalities established during chronic infection. Epigenetic remodeling, mitochondrial dysfunction, oxidative genomic injury, and chromatin-associated dysregulation may persist after bacterial clearance, particularly in patients with intestinal metaplasia or advanced atrophic gastritis [67,100].

Long-term investigations demonstrate persistence of methylation abnormalities involving CDH1, CDKN2A, MLH1, MGMT, and microRNA-associated regulatory networks despite successful eradication therapy [67,100]. These observations suggest that prolonged infection may establish durable epithelial alterations capable of influencing tissue behavior beyond removal of the initiating pathogen.

Residual cancer risk reflects more than bacterial persistence alone. Incomplete mitochondrial recovery, stem-cell adaptation, and long-standing epigenetic dysregulation may continue to influence gastric biology following eradication, thereby supporting the need for prolonged surveillance and risk-oriented clinical management in selected patients [67,100]. Accordingly, the proposed framework should be interpreted as complementary to eradication-based prevention rather than as a strategy to defer treatment, with the objective of identifying residual biological risk among individuals who continue to exhibit persistent mucosal alteration after successful eradication [100,101].

### 9.2. Selective Disruption of Oncogenic Activity

Therapeutic approaches based solely on generalized antioxidant administration have demonstrated limited long-term effectiveness because oxidative intermediates participate in both pathological and physiologically necessary cellular processes [9,17]. Current evidence supports selective modulation of oncogenic signaling systems associated with chronic *H. pylori* infection rather than indiscriminate suppression of oxidative activity.

Inflammatory and survival-associated pathways including NF-κB, STAT3, PI3K/AKT, HIF-1α, and NOX1 contribute to inflammatory amplification, apoptosis resistance, metabolic adaptation, and epithelial persistence within gastric tissue [102,103,104]. Experimental and early translational investigations suggest that selective interference within these systems may provide greater mechanistic specificity while limiting disruption of normal cellular regulation. Studies have explored ferroptosis-associated targeting, mitochondrial-directed modulation, selective oxidative regulation, and nanotherapeutic delivery systems. However, these approaches currently lack sufficient clinical validation for routine therapeutic application [17,80,105]. Although many remain in preclinical development, they suggest a shift toward mechanism-oriented therapeutic regulation rather than broad oxidative suppression.

### 9.3. Importance of Temporal and Compartment-Specific Intervention

Therapeutic effectiveness depends not only on target selection but also on disease stage, tissue context, and intracellular localization. Oxidative processes exert compartment-specific effects that vary according to inflammatory burden, mitochondrial function, epithelial organization, and histological progression. Excessive suppression may therefore interfere with epithelial integrity, immune coordination, and adaptive recovery mechanisms required for mucosal stability [9,104].

Clinical benefit appears greatest before advanced structural and regulatory abnormalities become established. Early eradication therapy may partially reverse inflammatory and methylation-associated alterations, whereas advanced intestinal metaplasia and dysplasia demonstrate substantially lower reversibility [67,100].

Intracellular localization remains important because epithelial cells, immune populations, stromal compartments, and mitochondria exhibit distinct oxidative behavior during chronic infection. Future therapeutic platforms may benefit from selective compartment-targeting strategies capable of interrupting malignant progression while preserving physiological tissue organization [17,19,105].

### 9.4. Integrated Precision-Oriented Therapeutic Frameworks

Future management of *H. pylori*-associated gastric disease will likely require integration of antimicrobial eradication with biologically guided modulation of inflammatory, metabolic, epigenetic, and oncogenic abnormalities. Although eradication therapy remains essential for removal of the initiating microbial stimulus, tissue-associated dysregulation may continue to influence cancer susceptibility following bacterial clearance [67,100].

Combination approaches integrating eradication therapy with selective intervention represent a potential future direction, although their long-term clinical benefit remains to be established. Investigational strategies under investigation include modulation of NF-κB- and STAT3-associated signaling, ferroptosis-related targeting, mitochondrial protection, epigenetic-directed intervention, and precision-oriented oxidative regulation designed to preserve tissue organization while limiting malignant progression [106,107]. At present, eradication therapy remains the only established intervention with proven population-level benefit for reducing *H. pylori*-associated gastric cancer risk [108].

Integrated clinical models may support individualized therapeutic planning according to histological stage, biological status, and molecular risk profile. Such precision-guided approaches could improve prevention of gastric injury and reduce long-term cancer susceptibility beyond conventional infection-centered management alone [109].

Representative clinical scenarios may help illustrate this framework. Patients with active *H. pylori* infection remain candidates for established eradication therapy and routine clinical follow-up [108]. In contrast, individuals with persistent atrophic or metaplastic alterations after eradication may benefit from risk-oriented surveillance and biomarker-informed assessment [100,101]. More advanced biological reprogramming states may represent potential settings for investigational precision-directed interventions once clinical validation becomes available [17].

Importantly, this framework should not be interpreted as an alternative to population-level *H. pylori* detection and eradication. Instead, it is intended to complement established prevention strategies by improving identification of individuals who continue to demonstrate residual biological risk despite appropriate microbial management [101,108].

**Figure 4:**
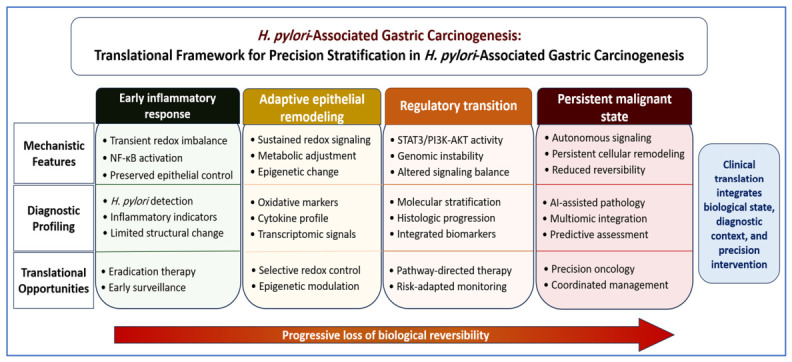
Translational framework for precision stratification in *H. pylori*-associated gastric carcinogenesis. The framework presents an integrated view linking biological state, diagnostic interpretation, and context-dependent translational opportunities across progressive loss of biological reversibility.

Figure 4 summarizes the proposed relationship among mechanistic stratification, diagnostic interpretation, and stage-dependent therapeutic intervention during *H. pylori*-associated gastric carcinogenesis.

## 10. Integrated Multidisciplinary Framework and Future Directions

### 10.1. Integrated Model of Infection-Associated Carcinogenesis

Current evidence suggests that chronic *H. pylori* infection promotes gastric carcinogenesis through coordinated interaction among inflammatory signaling, oxidative injury, metabolic adaptation, epigenetic remodeling, epithelial plasticity, and microenvironmental change rather than isolated molecular damage alone [16,110]. Within this framework, chronic inflammatory and metabolic stress alters epithelial behavior across molecular, cellular, and tissue levels during carcinogenic progression.

Instead of viewing oxidative stress solely as a source of molecular injury, this framework emphasizes its role in coordinating interactions among inflammatory, metabolic, epigenetic, and microenvironmental processes [9,110]. Oxidative stress, inflammation, metabolic remodeling, epigenetic alterations, and microenvironmental changes should not be viewed as independent processes. The proposed framework highlights how these events interact throughout disease progression. Redox signaling is presented as a common biological link that connects these alterations during chronic *H. pylori* infection and gastric carcinogenesis [17,38,66]. By integrating these interconnected processes within a single biological model, the framework provides a broader understanding of disease progression than considering each mechanism separately and offers a unified perspective for interpreting biological changes across the gastric carcinogenesis cascade. This perspective helps integrate findings that are often discussed separately and provides a foundation for more biologically informed diagnostic and therapeutic strategies.

Oxidative signaling serves as an important coordinating mechanism that influences multiple regulatory systems involved in epithelial adaptation during chronic infection. Reactive intermediates influence transcriptional regulation, mitochondrial function, chromatin organization, and cellular metabolism, thereby altering epithelial survival and proliferative behavior [9,19]. Over time, these alterations support emergence of epithelial phenotypes associated with malignant transformation.

This framework also incorporates emerging concepts involving stem-cell plasticity, spatial heterogeneity, microbiome-associated regulation, and adaptive signaling networks. Gastric carcinogenesis therefore appears to result from interaction among inflammatory, metabolic, epithelial, and microenvironmental processes throughout disease progression [110,111].

### 10.2. Testable Biological Transitions and Experimental Priorities

Current evidence strongly supports the involvement of oxidative stress, chronic inflammation, and DNA damage in *H. pylori*-associated gastric carcinogenesis [3,7,17]. However, important limitations remain. Much of the mechanistic evidence originates from experimental models, and the complexity of disease progression in humans is not yet fully understood [22,78]. Although increasing evidence links redox imbalance with metabolic remodeling, epigenetic alterations, and microbial dysbiosis, the temporal relationships among these processes remain incompletely defined [38,49,66]. In addition, several biomarker-based and molecular risk-stratification approaches have shown promising diagnostic and prognostic potential, but their clinical utility requires further validation in large prospective studies [87,94,98]. Hence, the threshold framework proposed in this review should be interpreted as a biologically informed conceptual model instead of a clinically validated classification system [13,17].

Future research should prioritize development of experimentally measurable models capable of identifying critical transition states during *H. pylori*-associated carcinogenesis [16]. At present, these transition states remain theoretical constructs that require prospective biological validation. Furthermore, much of the available evidence is derived from experimental and associative studies, and the temporal relationships linking individual molecular alterations to gastric cancer progression remain incompletely defined [16,110]. A major objective is distinguishing reversible epithelial alteration from biologically stabilized malignant behavior during chronic infection.

Potential measurable parameters include oxidative DNA injury, mitochondrial dysfunction, impaired antioxidant buffering, inflammatory activity, chromatin remodeling, transcriptional alteration, and metabolic reprogramming. Longitudinal evaluation of these variables may improve mechanistic stratification and facilitate earlier recognition of high-risk gastric states before irreversible transformation becomes established [112].

Further progress will likely depend on integration of multi-omics analysis, spatial profiling, organoid-based modeling, and computational prediction platforms. These approaches may clarify how interacting signaling pathways influence disease progression and may support predictive models linking molecular activity with clinically meaningful pathological change [110].

### 10.3. Translational Priorities

Future translational efforts should integrate mechanistic interpretation, diagnostic validation, predictive modeling, and precision-guided intervention more directly. An important priority is development of clinically applicable strategies capable of identifying high-risk gastric states before malignant transformation becomes biologically established [16,113].

Prospective investigations combining genomic profiling, inflammatory characterization, metabolic assessment, histopathological interpretation, and computational analysis may improve individualized risk evaluation and facilitate earlier identification of high-risk gastric disease. AI-assisted analysis and computational modeling may improve interpretation of complex biological interactions in both diagnostic and translational settings [97,110].

Therapeutic development should continue moving beyond generalized oxidative suppression toward selective regulation of disease-relevant signaling pathways and stage-dependent pathogenic processes. Precision-guided approaches integrating eradication therapy with selective targeting and risk-adapted surveillance may support more selective therapeutic approaches while preserving normal tissue function [16,17].

## 11. Conclusions

*H. pylori*-associated gastric carcinogenesis develops through interaction among inflammatory activity, oxidative dysregulation, metabolic alteration, epigenetic remodeling, and microenvironmental change rather than isolated oxidative injury alone. Persistent intracellular stress progressively alters epithelial regulation, promotes genomic instability, and favors emergence of abnormal cellular states associated with malignant transformation. This review supports a multidimensional framework in which disease progression reflects increasing biological instability together with declining reversibility during chronic infection. Current evidence additionally highlights limitations of detection-centered diagnostics and non-selective antioxidant strategies, thereby emphasizing the importance of biologically contextualized risk assessment and precision-oriented intervention. Future progress will likely depend on integrating mechanistic insight with predictive diagnostics, computational tools, and targeted therapeutic strategies to strengthen prevention, surveillance, and early management of *H. pylori*-associated gastric cancer.

## Figures and Tables

**Table 1:** Contrasting acute oxidative injury and chronic redox regulation in *H. pylori* infection.

Dimension	Acute Oxidative Injury	Chronic Redox Regulation in *H. pylori* Infection	Refs.
Trigger context	Short-lived immune activation	Persistent bacterial colonization with unresolved inflammation	[3,7]
Reactive species profile	High-amplitude bursts of ROS and RNS	Continuous low-to-moderate ROS and RNS generation	[18]
Primary sources	Activated neutrophils and macrophages	Immune cells, epithelial NOX1 activity, and mitochondrial ROS	[9,18]
Nitrosative contribution	Transient nitric oxide production	Sustained iNOS activation with peroxynitrite formation	[19]
Spatial distribution	Diffuse and non-specific	Compartmentalized at membrane, mitochondria, and immune interfaces	[9,29]
Temporal pattern	Rapid onset and resolution	Persistent exposure with repeated signaling cycles	[11]
Antioxidant response	Rapid consumption with limited adaptation	Partial depletion with maintained functional capacity	[12,26]
Mitochondrial involvement	Secondary damage under excessive stress	Continuous ROS generation with incomplete functional recovery	[9,30]
Signaling engagement	Minimal or stress-induced activation	Sustained activation of redox-sensitive signaling pathways	[24]
Feedback regulation	Weak or absent	NOX-dependent amplification loops maintaining ROS production	[24]
Cellular outcome	Cytotoxicity, necrosis, or apoptosis	Adaptation with preserved viability and altered function	[10]
Metabolic state	Disruption of cellular metabolism	Shift toward glycolytic support under oxidative conditions	[30]
Immune interaction	Pathogen clearance-oriented	Sustained inflammatory signaling and immune modulation	[17]
Tissue effect	Acute injury with potential recovery	Long-term mucosal alteration under persistent inflammation	[3]
Biological role	Damage-driven response	Signaling-associated regulatory system	[11]
Reversibility	Often reversible after insult removal	Maintained state due to ongoing infection	[7]
Disease implication	Limited long-term impact if resolved	Supports chronic pathology and predisposes to carcinogenesis	[8]

**Table 2:** Redox-sensitive pathways coordinating gastric epithelial transformation during chronic *H. pylori* infection.

Pathway/System	Redox-Associated Regulation	Major Effects	Coordinated Interactions	Refs.
NF-κB	ROS-mediated inflammatory activation	Cytokine production, inflammatory persistence	Interacts with STAT3 and HIF-1α signaling	[32,53]
STAT3	Cytokine-associated activation reinforced by oxidative conditions	Survival, proliferation, angiogenesis	Integrates with NF-κB and PI3K/AKT pathways	[51,56]
HIF-1α	Oxidative stabilization during chronic stress	Metabolic adaptation, epithelial survival	Connects inflammatory and metabolic regulation	[37,71]
PI3K/AKT	Oxidative activation of upstream signaling	Cell survival, metabolic adaptation, cell-cycle progression	Cooperates with MAPK and STAT3 pathways	[34,64]
MAPK/ERK	CagA-associated and oxidative pathway activation	Proliferation and epithelial remodeling	Functions with PI3K/AKT and NF-κB pathways	[34,55]
Wnt/β-catenin	β-catenin stabilization induced by oxidative signaling and CagA	Epithelial transition, proliferation, stemness-associated activity	Interacts with Hippo-YAP signaling	[1,57]
Hippo-YAP	Redox-sensitive YAP activation during chronic inflammation	Invasion, proliferation, epithelial remodeling	Cooperates with β-catenin and STAT3 pathways	[17,57]
DNA repair/checkpoint systems	Oxidative DNA injury with impaired repair activity	Mutation accumulation, genomic instability	Linked to mitochondrial dysfunction and epigenetic dysregulation	[59,62]
Epigenetic regulation	Oxidative modulation of methylation and chromatin remodeling	Stable transcriptional dysregulation and tumor suppressor silencing	Reinforces inflammatory and proliferative pathways	[40,66]
Mitochondrial redox-associated regulation	Persistent mitochondrial ROS production	Metabolic alteration, DNA damage, survival regulation	Supports inflammatory and epigenetic remodeling	[9,59]

**Table 3:** Antioxidant strategies versus system-level consequences and mechanistic limitations.

Antioxidant Strategy	Intended Outcome	System-Level Effect	Principal Limitation in *H. pylori*-Associated Carcinogenesis	Refs.
Broad reactive-species scavenging	Lower oxidative burden	Loss of physiological redox activity	Cannot distinguish damaging oxidative injury from processes required for epithelial and immune homeostasis	[9,12]
Non-specific antioxidant supplementation	Reduce inflammatory injury	Variable biological response	Therapeutic effects depend on timing, intracellular localization, and oxidative intensity	[16,17]
Prolonged antioxidant exposure	Maintain long-term redox suppression	Impaired adaptive stress responses	May interfere with mitochondrial resilience and epithelial survival pathways	[9,80]
Global inhibition of redox-sensitive pathways	Limit inflammatory activation	Disruption of signaling network integration	Alters kinase activity and transcriptional organization beyond pathological processes	[9,12]
Non-targeted antioxidant delivery	Neutralize intracellular oxidants	Weak compartment-specific activity	Insufficient control of localized redox regulation during epithelial transformation	[9,16]
Untargeted anti-inflammatory antioxidant therapy	Decrease chronic inflammation	Persistence of oncogenic activity	Does not fully interrupt integrated carcinogenic circuitry	[16]
Precision redox modulation	Selectively regulate pathogenic oxidative signaling	Preservation of cellular homeostasis	Requires precise spatial and temporal control	[1,25]
Redox-directed nanotherapeutics	Improve intracellular selectivity	Reduced disruption of homeostatic pathways	Clinical translation remains limited by delivery complexity	[81,82]
Pathway-selective oxidative intervention	Modulate defined oncogenic circuits	Greater mechanistic specificity	Requires detailed mapping of dynamic redox interactions	[1,17]
Selective redox cycling against *H. pylori*	Exploit bacterial oxidative vulnerability	Increased antimicrobial selectivity	Long-term host-pathway specificity remains incompletely defined	[83]

## Data Availability

No new data were created or analyzed in this study.
